# Pretreatment Nutritional Status in Combination with Inflammation Affects Chemotherapy Interruption in Women with Ovarian, Fallopian Tube, and Peritoneal Cancer

**DOI:** 10.3390/nu14235183

**Published:** 2022-12-06

**Authors:** Naoko Nomoto, Shinichi Tate, Makoto Arai, Shinji Iizaka, Chisato Mori, Kenichi Sakurai

**Affiliations:** 1 Department of Nutrition and Metabolic Medicine, Graduate School of Medical and Pharmaceutical Sciences, Chiba University, Chiba 260-8670, Japan; 2 Department of Clinical Nutrition, Chiba University Hospital, Chiba 260-8677, Japan; 3 Division of Gynecology, Chiba University Hospital, Chiba 260-8677, Japan; 4 Department of Medical Oncology, Chiba University Hospital, Chiba 260-8677, Japan; 5 School of Nutrition, College of Nursing and Nutrition, Shukutoku University, Chiba 260-8701, Japan; 6 Department of Sustainable Health Science, Center for Preventive Medical Sciences, Chiba University, Chiba 263-8522, Japan; 7 Department of Bioenvironmental Medicine, Graduate School of Medicine, Chiba University, Chiba 263-8522, Japan; 8 Department of Nutrition and Metabolic Medicine, Center for Preventive Medical Sciences, Chiba University, Chiba 263-8522, Japan

**Keywords:** nutritional status, chemotherapy interruption, ovarian cancer, fallopian tube cancer, peritoneal cancer

## Abstract

Background: Discontinuing chemotherapy worsens cancer prognosis. This study aimed to investigate the relationship between nutritional status at the start of chemotherapy and chemotherapy discontinuation in patients with ovarian, fallopian tube, and primary peritoneal cancer. Methods: This was a retrospective cohort study. One hundred and forty-six patients to whom weekly paclitaxel and carboplatin were administered as postoperative chemotherapy were included. Six courses in 21-day cycles were defined as complete treatment. As nutritional indicators, body mass index, weight change rate, serum albumin, total lymphocyte count, prognostic nutritional index, and C-reactive protein-to-albumin ratio (CAR) were compared between complete and incomplete treatment groups. Patients were divided into two groups according to CAR. The number of chemotherapy cycles was compared between these two groups. A Cox proportional hazard model was used for covariate adjustment. Results: Several indicators differed between complete and incomplete treatment groups, and among the indicators, CAR had the highest discriminatory ability. The number of chemotherapy cycles was shorter in the high CAR group than in the low CAR group. A high CAR was associated with chemotherapy interruption even after adjusting for covariates. Conclusion: Based on CAR, nutritional status before chemotherapy is suggested to be associated with the risk of chemotherapy discontinuation.

## 1. Introduction

Cancer is one of the leading causes of morbidity and mortality worldwide [1]. The incidence and mortality of cancer are increasing rapidly. In 2020, 19.3 million cases and 10 million cancer deaths were estimated worldwide. Approximately half of these occurred in Asia, accounting for 58.3% of the global cancer mortality rate [2]. The outcomes of cancer treatments such as surgery, radiation therapy, and chemotherapy have improved owing to medical advances [3,4]. Advances in surgical therapy, including robotic surgery, has been applied and enables precision surgery [5,6,7]. The introduction of molecular targeted therapies such as anti-angiogenesis agents including vascular endothelial growth factor (VEGF) antibodies and receptor tyrosine kinase (RTK) inhibitors has resulted in improved therapeutic response and reduced systemic toxicity [8]. To analyze circulating DNA (liquid biopsy), immune markers, and other biological features of the patients in order to evaluate the efficacy of anti-cancer agents and make treatment decisions, precision medicine approaches have been taken [9]. Treatment with new chemotherapy drugs extended overall survival [4].

Chemotherapy is classified into four categories, first-line chemotherapy, neoadjuvant chemotherapy (NAC), maintenance chemotherapy, and secondary chemotherapy. First-line chemotherapy is the first chemotherapy aimed at improving treatment outcomes and is performed postoperatively. NAC is preoperative chemotherapy performed to improve the completion rate of radical surgery. Maintenance chemotherapy is a treatment performed for long-term survival after remission. Secondary chemotherapy is chemotherapy in the event of a relapse or resistance to initial chemotherapy. The prognosis of several cancers has been reported to improve in cases where chemotherapy is fully completed [10]. However, chemotherapy causes several side effects, such as blood and lymphatic disorders, cardiac disorders, eye disorders, gastrointestinal disorders, immune system disorders, infectious and general nervous system disorders, mental disorders, renal and urinary disorders, and others. In severe cases of chemotherapy side effects, it can be life-threatening [11]. In addition, the chemotherapy interruption rate due to the side effects ranges from 10 to 70% [12,13,14] and has been associated with a worse prognosis [10,15]. Therefore, it is necessary to clarify the factors contributing to chemotherapy discontinuing and implement countermeasures. 

Ovarian cancer is the second most common gynecological cancer worldwide among women [16]. The age-adjusted incidence of the serous ovarian, fallopian tube, and peritoneal cancers is 4.86%, 0.63%, and 0.62%, respectively, in the United States [17]. These three cancers have common histological and clinical features [18,19]. Therefore, in the 2014 International Federation of Gynecology and Obstetrics (FIGO) classification, the three cancers are considered to share many clinical and morphologic similarities [20]. Treatment for ovarian, fallopian tube, and peritoneal cancer is a multidisciplinary treatment based on surgical therapy and is mainly combined with chemotherapy. In recent years, molecularly targeted drugs have also been used. Chemotherapy for ovarian cancer has progressed over recent decades and has improved treatment outcomes [21,22,23,24,25]. Currently, the standard of care first-line chemotherapy for ovarian, fallopian tube and peritoneal cancer includes paclitaxel and carboplatin. Weekly first-line chemotherapy with cisplatin and carboplatin for these cancers has been reported to be completed by 62% with the occurrence of anemia, constipation, fatigue, decreased neutrophil count, low platelet count, peripheral sensory neuropathy, and various other side effects [25].

Nutritional status plays an important role in treating various diseases, and cancer is no exception. Nutritional interventions are known to improve the results of cancer treatment as an adjunctive therapy [26]. The preoperative nutritional status reportedly affects some cancers’ treatment results and prognosis [27,28]. Cousin et al., reported that the pre-chemotherapy nutritional status affected the development of side effects of chemotherapy [29]. Bougnoux et al., reported that a nutritional intervention during chemotherapy reduced the mortality rate in female patients with a median age of 58 years [30]. In these reports, they examined several nutritional indicators, such as body mass index (BMI) [26], weight change rate [31], serum albumin level [28], the Skeletal Muscle Index [32], the Nutritional risk Screening 2002 [33]. There are also other nutritional indicators. Total lymphocyte count (TLC) [34,35], prognostic nutritional index (PNI) [36], the controlling nutritional status [37], and C-reactive protein-to-albumin ratio (CAR) [38] are used as a tool to screen for undernutrition in hospitalized patients. In addition, the Global Leadership Initiative on Malnutrition (GLIM) criteria has been proposed to indicate the severity of nutritional status. It was created to adopt global consensus standards so that malnutrition’s prevalence, interventions, and outcomes can be compared worldwide [39].

Although nutritional status influences the course of chemotherapy, little data are available on the association between nutritional status and chemotherapy discontinuation [40]. In addition, most patients with advanced ovarian cancer have a high frequency of peritoneal metastases. Because of this, abdominal pain, bloating, loss of appetite, vomiting symptoms, and intestinal obstruction are easy to complicate, food intake decreases, and nutritional status deteriorates. According to Hertlein et al., 35.8% of hospitalized patients with ovarian cancer are at risk of malnutrition [27]. Even in patients with ovarian cancer prone to malnourishment, there are only a few studies on the association between nutritional status and chemotherapy interruption [41,42]. If we can anticipate chemotherapy interruptions owing to worsened nutritional status and intervene preemptively, we may be able to improve treatment continuity. Therefore, we aimed to investigate the relationship between nutritional status at the start of chemotherapy and chemotherapy discontinuation in ovarian, fallopian tube, or peritoneal cancer patients. 

## 2. Materials and Methods

### 2.1. Participants

We extracted patients from the clinical database of Chiba University Hospital. The inclusion criteria were being diagnosed with ovarian, fallopian tube, or peritoneal cancer; and receiving weekly paclitaxel and carboplatin (WTC) therapy as postoperative chemotherapy after staging laparotomy, primary debulking surgery, or exploratory laparotomy. Staging was performed according to the International Federation of Gynecology and Obstetrics (FIGO) 2014 classification [20].

The exclusion criteria were receiving preoperative chemotherapy, recurrent cancer, having surgery at another hospital, history of chemotherapy, and coexistence of unknown cancers. 

### 2.2. Ethics

This study was performed in accordance with the Declaration of Helsinki and other national regulations. The study protocol was approved by the ethics committee of the Graduate School of Medicine, Chiba University. Informed consent was obtained in the form of opt-out, in line with information posted on the bulletin boards in the hospital. 

### 2.3. Study Design

We analyzed the records of all patients who received postoperative WTC for ovarian, fallopian tube, or peritoneal cancer between April 2016 and March 2021.

All the patients received WTC therapy; carboplatin was administered at an area under the curve (AUC) of 2 from Calvert’s formula and paclitaxel at 80 mg/m^2^ weekly. The scheduled regimen for the systemic WTC was 1, 8, and 15 over a 21-day cycle for six cycles. The total number of administrations was eighteen; accordingly, this was used to define the completion of chemotherapy. The group that received chemotherapy 18 times was defined as the complete treatment group and the group that did not complete treatment was defined as the incomplete treatment group. 

Heights and weights were measured by nurses. Blood and biochemical tests were performed at the Division of Laboratory Medicine, Chiba University Hospital. White blood cell count (WBC), hemoglobin level (HGB), and lymphocyte ratio were assessed by XN2000 (Sysmex Inc. Kobe, Japan) and biochemical analysis was performed by JCA-BM8040 (JEOL Ltd. Tokyo, Japan). 

As indicators of nutritional assessment, we assessed Onodera’s PNI, ALB, HGB, TLC, and CAR before chemotherapy. The TLC was calculated using the following formula: TLC = WBC × lymphocyte (%). The PNI was calculated using the following formula: PNI = 10 × ALB + 0.005 × TLC. Weight change rate was calculated as (weight before the first course of chemotherapy − weight before surgery)/(weight before surgery) × 100%. 

### 2.4. Statistical Analysis

As appropriate, clinicopathological parameters were compared using the Mann–Whitney U test, chi-square test, or Fisher’s exact test. Using receiver operating characteristic curve analysis, we compared the discriminatory ability of BMI, weight change rate, TLC, ALB, PNI, and CAR to detect patients who did not complete WTC. The AUC of each indicator was compared, and the indicator with the maximum values was used for survival analysis. The cut-off value was determined using the maximum Youden index, calculated as sensitivity + specificity − 1.

We divided the patients into two groups using these cut-off values and compared their treatment times using Kaplan–Meier analysis and the log-rank test. A Cox proportional hazards model was used for the univariate and multivariate analyses of chemotherapy interruption. We used a multivariate Cox proportional hazards model to control for the potential covariant roles of age, body mass index (BMI), weight change rate, TLC, CRE, and alanine aminotransferase as continuous variables, and tumor stage (I–II/III–IV) as a binary variable. CAR was included as a binary variable with a cut-off value. Additionally, stratified analyses by tumor stages were performed.

A power analysis was conducted to determine the sample size and power of at least 0.8 at a significance level of α = 0.05. 

Statistical analyses were performed using the JMP Pro statistical discovery software version 15.0 (SAS Institute, Cary, NC, USA). Statistical significance was set at 0.05.

## 3. Results

**Figure 1:**
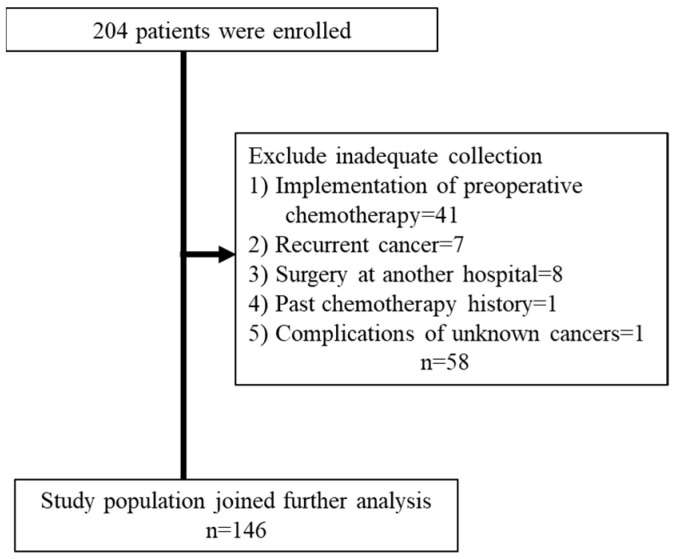
Participants selection flowchart.

In total, 204 patients were included in the database. Among them, we excluded 41 patients who had received preoperative chemotherapy, seven with recurrent cancer, eight who underwent surgery at another hospital, and one with previous chemotherapy. Furthermore, one patient with an unknown cancer was excluded. Accordingly, 146 patients were included in this study (Figure 1). 

We used our pilot study data for the power analysis, with a mean difference of ALB of 2.3 (standard deviation of 4.0) between the complete and incomplete treatment groups. Based on the results of the pilot data, the estimated sample size was 49 in each group and we achieved a sufficient number.

Patient characteristics are shown in Table 1. The median age was 59.5 years old. There were five patients in tumor stage I, 13 patients in stage II, 88 patients in stage III, and 40 patients in stage IV. The proportion of patients who used bevacizumab in combination during WTC treatment was 38% of all subjects. Of all patients, 85% had ascites. Eighty-three patients completed chemotherapy (complete treatment group), and 63 did not complete chemotherapy (incomplete treatment group). Age, body weight, aspartate aminotransferase, and CRP levels of the incomplete treatment group were significantly higher than those of the complete treatment group. Tumor stage also differed between the two groups. The most common reason for discontinuation in the incomplete treatment group was drug-related side effects, such as neutropenia, thrombocytopenia, and drug allergy. 

We compared nutritional indicators between the complete and incomplete treatment groups (Table 2). The incomplete treatment group showed significantly lower ALB, TLC, and PNI values than the complete treatment group. The BMI and CAR of the incomplete treatment group were significantly higher than those of the complete treatment group. The other factors did not differ between the groups. 

**Figure 2:**
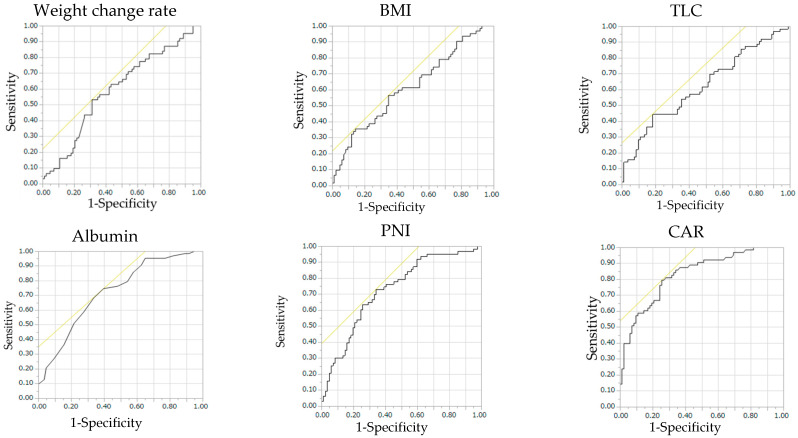
ROC curve of chemotherapy interruption for each nutritional indicator. BMI, body mass index; TLC, total lymphocyte count; PNI, prognostic nutritional index; CAR, C-reactive protein-to-albumin ratio. Sensitivity and specificity for interruption of chemotherapy were calculated using the cut-off values.

The ROC curve of each nutritional index for chemotherapy interruption is shown in Figure 2. ALB, PNI, and CAR showed a moderately high AUC in predicting chemotherapy interruption. The CAR had the largest AUC and a cut-off value of 0.24, corresponding to a sensitivity and specificity of 0.79 and 0.74, respectively (Table 3). Therefore, we used the cut-off value for CAR to divide the patients into low (74 patients) and high CAR (71 patients) groups. 

**Figure 3:**
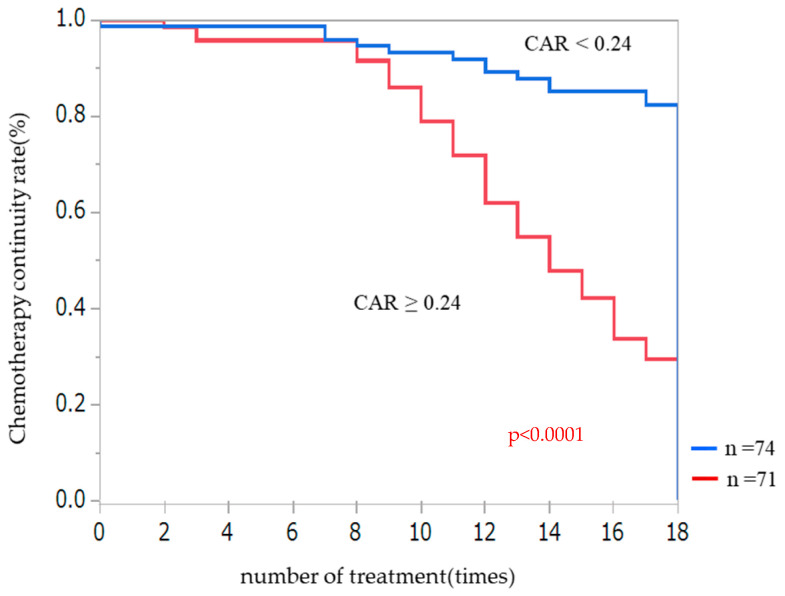
Kaplan–Meier curves of chemotherapy continuation according to the CAR. Patients were divided into two groups with 0.24 as the cut-off value. CAR: C-reactive protein-to-albumin ratio.

Chemotherapy was discontinued earlier in the group with a high CAR than in the group below the cut-off value (Figure 3). Eighty-two percent of patients in the low CAR group completed chemotherapy, whereas the same was true only for 30% of patients in the high CAR group. Using the Cox proportional hazard model, we found that a high CAR was significantly associated with chemotherapy interruption both in the adjusted and unadjusted models. The HR of the high CAR group compared with the low CAR group was 1.84 (95% CI 1.26–2.69, *p* < 0.01) (Table 4).

Furthermore, stratified analysis by tumor stages (I–II/III–IV) showed a similar tendency for the CAR group; the HR of the high CAR group was 1.73 (95% CI 1.17–2.54, *p* < 0.01) for stages III–IV (*n* = 125) and 23.8 (95% CI 0.71–794.17, *p* = 0.068) for stages I–II (*n* = 18). In stages I–II, the result was not significant due to the small sample size. 

## 4. Discussion

In this study, patients who did not complete chemotherapy had a worse nutritional status, with the CAR showing the highest sensitivity and specificity for predicting chemotherapy interruption. Patients with high CAR showed a higher risk of chemotherapy interruption. 

Nutritional status is thought to be an important factor in cancer treatment, care, and prognosis [43,44]. In our study, several nutritional indicators showed a relationship with the discontinuation of chemotherapy. Of these, the CAR was the most predictive factor. CAR has been used as a predictive marker for mortality in acute medical admissions [45] and has been reported to be a predictor of tumor prognosis and perioperative risk [46]. CAR includes CRP and ALB levels in its calculation. A high CAR indicates high CRP and low ALB levels. Serum albumin level is one of the nutritional indicators. Serum albumin level is affected by food intake, intestinal absorption, inflammatory condition, and other factors [47,48]. Dietary protein is a sauce of albumin synthesis in a liver. Patients with ovarian, fallopian tube, and peritoneal cancer frequently have ascites which can cause abdominal distension and appetite loss. Decreased dietary intake caused by these situations can reduce albumin synthesis and serum albumin level. High CAR can partly represent decreased nutritional status. Thus, our results suggest the association between chemotherapy interruption and nutritional status. Serum albumin is a nutritional assessment indicator for clinically stable people [47]. Malafarina et al., reported that blood levels of serum albumin do not depend on nutritional status alone, so they may be an excellent nutritional indicator when considering inflammatory status [49]. According to Akahori et al., the PNI is lower in the failure-to-complete treatment group than in the complete treatment group of adjuvant chemotherapy in patients with pancreatic cancer [40]. They reported that a lower preoperative PNI was associated with failure to complete chemotherapy. Aaldriks et al., reported that poor nutrition increases the probability of uncompleted chemotherapy for some chemotherapy regimens for different types of cancers [42]. These are consistent with our study findings and suggest that nutritional status and chemotherapy interruption are related. In addition, according to Phippen et al., the group with neutropenia, a side effect of chemotherapy in ovarian cancer, was reported to have a lower nutritional status than the group without neutropenia [50]. Furthermore, undernutrition affects the pharmacokinetics of several anticancer agents [51,52,53]. 

Cancer frequently causes systemic inflammation. Severe inflammatory status is associated with fever, appetite loss, body weight loss, exhaustion, and other physical symptoms. Systemic inflammation shows many changes in biochemical analysis, such as CRP. Serum levels of inflammatory cytokines are elevated including interleukin-6, interferon gamma, tumor necrosing factor-alpha, interleukin-8, interleukin-18 and others [54]. Elevated white blood cell count is also observed. Cytokines are important regulators of inflammation and are involved in acute and chronic inflammation through a network of interactions. Pro-inflammatory chemokines are produced by cells to mobilize leukocytes to the site of infection or injury [55]. Inflammation causes a decrease in nutritional status by inducing catabolism and loss of appetite. It is reported that serum albumin level is affected by systemic inflammation [56]. Albumin synthesis in a liver is inhibited by inflammation [57]. In the inflammatory status, albumin is required as a material to produce other proteins in peripheral tissues, and serum albumin level is reduced by the consumption [54]. In our study, serum albumin level was possible to be affected by inflammation on cancer bringing condition. Serum albumin levels of the patients in this study could reflect their inflammatory conditions.

Inflammation is also reported to be involved in developing the side effects of chemotherapy [58]. Chemotherapy-induced toxicity of cisplatin has been reported to be enhanced by inflammation [59,60]. Therefore, the presence of inflammation before chemotherapy may influence chemotherapy discontinuation due to the side effects of the anticancer drugs. In addition, inflammation causes physical and mental exhaustion [61,62], which can cause chemotherapy interruption in the case of malignancy. In our study, chemotherapy was discontinued due to fatigue in several cases. CAR is thought to be an indicator not only nutritional status, but also inflammatory condition. Therefore, the presence of inflammation before chemotherapy may influence chemotherapy discontinuation due to the side effects of the anticancer drugs. Although further studies are needed to clarify the mechanism of chemotherapy discontinuation due to undernutrition and inflammation, our results suggest that nutritional status and inflammation are involved in the interruption of cancer chemotherapy.

Recently, GLIM criteria have been proposed as an indicator of the severity of nutritional status. GLIM criteria consist of a two-stage model. The first step is malnutrition risk screening to identify the risk of poor nutritional status. The diagnosis of malnutrition and the assessment of severity grading follows this. First-step malnutrition risk screening indicators include Nutrition Risk Screening-2002 (NRS-2002), Mini Nutrition Assessment-Short Form (MNA-SF), Malnutrition Universal Screening Tool (MUST), and Subjective Global Assessment (SGA). In the second step, three are phenotypic criteria (weight loss, low body mass index, and reduced muscle mass), and two are etiologic criteria (reduced food intake or assimilation, and inflammation or disease burden). Diagnosing malnutrition requires at least one phenotypic criterion and one etiological criterion. At the next step, the severity of malnutrition is assessed based on a phenotypic criterion. The advantage of using the GLIM criteria is that the global consensus standard allows us to compare the prevalence, intervention, and outcomes of malnutrition worldwide. In this study, the GLIM criteria could not be applied for the following reasons: This study is a retrospective study, and the first step, nutritional screening, was not performed at the start of chemotherapy. There was no data on weight loss because the patients’ body weight before the cancer diagnosis was not measured. Their muscle mass was not assessed routinely in our hospital. Food intake before the first chemotherapy could not be assessed. GLIM criteria have been proposed as the global standard. Thus, it is necessary to plan our future study using GLIM criteria [39] for nutritional assessment. 

Usually, low BMI represents a deterioration of the nutritional status [63]. In this study, most nutritional indicators showed an association between low nutritional status and chemotherapy interruption, as seen in previous studies, with BMI. Most of our results are consistent with those of previous studies [40]; however, the complete treatment group showed lower BMI than the incomplete treatment group, indicating results opposite to that of a previous study [29]. In addition, patients with renal cell carcinoma and low BMI have been reported to have a high frequency of chemotherapy side effects [64]. These different outcomes may be due to the effects of ascites since ovarian, fallopian tube, and peritoneal cancers have been reported to be prone to ascites-associated complications [28,65,66]. Our results indicate that it is difficult for patients with these three cancers to use BMI for nutritional assessment.

In the present study, CAR was suggested as a predictor of chemotherapy interruption, indicating that nutrition and inflammation play an important role. Nutritional interventions have been reported to improve nutritional status and inflammation [67]. Fish oil omega-3 polyunsaturated fatty acids, docosahexaenoic acid (DHA), and eicosapentaenoic acid (EPA), can reduce inflammation. In a randomized clinical trial involving 22 patients with hematological malignancies treated with chemotherapy, an improvement in the CAR was reported in the group receiving 2 g/day EPA and DHA for nine weeks [68]. It has also been reported that patients with ovarian cancer who receive a nutritional intervention using oral enteral nutrition have higher ALB levels during chemotherapy [69]. These findings suggest that nutritional interventions can improve the nutritional and inflammatory status and result in the continuation of chemotherapy for ovarian, fallopian tube, and peritoneal cancer. 

The strength of our research is that we were able to achieve the required sample size for sufficient statistical power. Secondly, all cases were postoperative and could withstand the surgery. Therefore, the physical conditions were optimal to some extent. Thirdly, the number of doses was fixed. Therefore, the use of a predetermined chemotherapy regimen made it possible to identify a more accurate association between chemotherapy and nutritional status. Despite these strengths, this study also had several limitations. This study reported the result of only one chemotherapy regimen for these tumors. Therefore, the results cannot be generalized to other cancers or chemotherapy regimens. We also included a limited number of nutritional indicators owing to the retrospective study design. We were not able to examine nutritional indicators that were not measured in this study. Especially, information of food intake was not obtained in this study, which is an important indicator for nutritional assessment. There may have also been potential effects of unmeasured confounding. In addition, we included patients from one hospital, which may have caused selection bias. A multicenter study is required to confirm these results. 

## 5. Conclusions

In conclusion, nutritional status before chemotherapy is associated with the risk of chemotherapy discontinuation. CAR is a useful nutritional indicator for predicting the discontinuation of chemotherapy. 

## Figures and Tables

**Table 1:** Characteristics of patients.

Characteristic	All Patients (*n* = 146)	Complete Treatment Group (*n* = 83)	Incomplete Treatment Group (*n* = 63)	*p*-Value
	Median (IQR)	Median (IQR)	Median (IQR)	
Age (years)	59.5 (49.8–69.0)	55.0 (46.0–63.0)	67.0 (54.0–71.0)	<0.01
Height (cm)	155.3 (151.3–159.2)	156.0 (151.3–159.0)	155.0 (151.3–159.8)	0.86
Body weight (kg)	51.7 (45.6–57.0)	50.0 (44.0–56.1)	52.6 (48.0–59.5)	0.02
AST (mg/dL)	19 (15–26)	18 (15–23)	20 (15–29)	0.04
ALT (mg/dL)	15.5 (11.0–24.3)	17 (11–25)	13 (10–23)	0.30
CRE (mg/dL)	0.54 (0.46–0.61)	0.53 (0.45–0.62)	0.54 (0.48–0.60)	0.86
WBC (/μL)	5.6 (4.8–7.0)	5.5 (4.6–6.6)	5.8 (4.8–7.7)	0.17
HGB (mg/dL)	10.7 (9.9–11.6)	10.8 (10.1–11.6)	10.5 (9.8–11.5)	0.42
CRP (mg/dL)	0.7 (0.1–3.0)	0.2 (0.1–1.0)	2.9 (0.9–7.0)	<0.01
Tumor stage I–II/III–IV	I–II 18/III–IV128	I–II 15/III–IV 68	I–II 3/III–IV 60	0.02
Treatment with bevacizumab, *n* (%)	55 (37.7)	36 (43.4)	19 (30.2)	0.10

Data are presented as median (IQR, interquartile range). Tumor stage is expressed as the number of patients included in the two groups, stages I–II or III–IV. AST, aspartate aminotransferase; ALT, alanine aminotransferase; CRE, creatinine; WBC, white blood cell count; HGB, hemoglobin; CRP, C-reactive protein. Tumor stage classification was performed according to International Federation of Gynecology and Obstetrics 2014, metastasis classification. The complete treatment group completed 18 times of chemotherapy; the incomplete treatment group did not complete 18 times of chemotherapy. Statistical tests were performed using Mann–Whitney U test, Chi-Square test, or Fisher’s exact test.

**Table 2:** Comparison of nutritional indicators between the complete and incomplete treatment groups.

Nutritional and Inflammation Indicators	Complete Treatment Group (*n* = 83)	Incomplete Treatment Group (*n* = 63)	*p*-Value
	Median (IQR)	Median (IQR)	
BMI (kg/m^2^)	20.8 (18.7–23.9)	22.2 (19.4–25.1)	0.02
Albumin (g/dL)	3.3 (2.9–3.9)	2.8 (2.6–3.2)	<0.01
Weight change rate (%)	−4.5 (−8.6–0)	−2.1 (−5.6–+0.7)	0.05
TLC (/μL)	1050 (829–1475)	913 (686–1274)	0.01
PNI	54.2 (48.1–65.3)	47.1 (42.9–51.6)	<0.01
CAR	0.06 (0.02–0.31)	1.02 (0.34–2.42)	<0.01

Data are presented as median (IQR, interquartile range). BMI, body mass index; weight change rate (weight before the first course of chemotherapy—Weight before surgery)/(weight before surgery) × 100; TLC, total lymphocyte count; PNI, prognostic nutritional index; CAR, C-reactive protein-to-albumin ratio; complete treatment group completed 18 courses of chemotherapy; incomplete treatment group did not complete 18 courses of chemotherapy. Statistical tests were performed using Mann–Whitney U test.

**Table 3:** Sensitivity, specificity, AUC, and cut-off values of nutritional indicators for chemotherapy interruption.

Nutritional and Inflammation Indicators	AUC	*p*-Value	Cut-Off-Value	Sensitivity	Specificity
Weight change rate (%)	0.594	0.05	<2.35/≥2.35	0.69	0.53
BMI (kg/m^2^)	0.616	0.01	≥21.97/<21.97	0.65	0.56
TLC (/μL)	0.624	0.01	≥783.3/<783.3	0.82	0.44
Albumin (g/dL)	0.722	<0.01	≥3.1/<3.1	0.60	0.75
PNI	0.727	<0.01	≥49.95/<49.95	0.66	0.73
CAR	0.828	<0.01	<0.24/≥0.24	0.79	0.74

BMI, body mass index; TLC, total lymphocyte count; PNI, prognostic nutritional index; CAR, C-reactive protein-to-albumin ratio; AUC, area under the receiver operating characteristic (ROC) curve of the interruption of chemotherapy courses. The AUC was derived from the ROC analysis. The cut-off value determined the point at which the sensitivity and specificity were maximized. Sensitivity and specificity for interruption of chemotherapy were calculated using the cut-off values.

**Table 4:** Hazard ratio of chemotherapy interruption according to CAR.

Variables	Crude Model		Adjusted Model	
	HR (95% CI)	*p* Value	HR (95% CI)	*p*-Value
CAR (greater than)	2.06(1.48–2.87)	<0.01	1.84(1.26–2.69)	<0.01

Values are expressed as hazard ratio (HR) and 95% confidence interval (CI). CAR, C-reactive protein to albumin ratio. Patients were divided into two groups with cut-off value of CAR. Cox proportional hazard model was performed with or without adjustment for age, Body mass index (BMI), weight change rate, total lymphocyte count (TLC), creatinine (CRE), and alanine aminotransferase (ALT) as continuous variables, and stages (I–II/III–IV) as a binary variable.

## Data Availability

Not applicable.
